# A Treatise on the Practice of Medicine

**Published:** 1886-12

**Authors:** 


A TREATISE ON THE PRACTICE OF MEDICINE. *For the use of Students and Practitioners of Medicine. By* ROBERTS BARTHOLOW, M.A., M.D., LL.D., *Professor of Materia Medica, General Therapeutics and Hygiene in the Jefferson Medical College of Philadelphia, etc., etc. Sixth Edition, Revised and Enlarged ; pp. 990. New York:* D. APPLETON & COMPANY. *Chicago:* A. C. MCCLURG & COMPANY. 1886.

In the preface to this edition the author anticipates adverse criticism by announcing, first, that this volume is only one of three which he intends to publish upon the subject of special pathology and therapeutics, and second, that he makes no claim to completeness for his treatment of the various subjects in the book. It is his intention that the treatment of the subjects taken up shall be eminently clinical rather than scientific, and that what he says shall represent his opinions rather than the opinions of other men.

The author says : "In the present edition some new subjects have been introduced, and preliminary chapters have been appended to the chief divisions of the work, to make the study of the diseases of the class more exact."

As a treatise on the practice of medicine the book has two grave faults. Too little attention is given to the history and pathology of the diseases discussed, and the treatment given represents too exclusively that of the author. And it is hardly enough for him to say in reply that what this book does not contain the other two will. In spite of its faults the book is a fair clinical guide as far as it goes, and the positive nature of the opinions expressed will commend it to some who like to be told just exactly what to prescribe in a given case.

